# Multiplex serology for impact evaluation of bed net distribution on burden of lymphatic filariasis and four species of human malaria in northern Mozambique

**DOI:** 10.1371/journal.pntd.0006278

**Published:** 2018-02-14

**Authors:** Mateusz M. Plucinski, Baltazar Candrinho, Geraldo Chambe, João Muchanga, Olinda Muguande, Graça Matsinhe, Guidion Mathe, Eric Rogier, Timothy Doyle, Rose Zulliger, James Colborn, Abu Saifodine, Patrick Lammie, Jeffrey W. Priest

**Affiliations:** 1 Division of Parasitic Diseases and Malaria, Centers for Disease Control and Prevention, Atlanta, United States of America; 2 President’s Malaria Initiative, Centers for Disease Control and Prevention, Atlanta, Georgia, United States of America; 3 National Malaria Control Program, Ministry of Health, Maputo, Mozambique; 4 Mozambique Field Epidemiology and Laboratory Training Program, Maputo, Mozambique; 5 Field Epidemiology and Laboratory Training Program, Centers for Disease Control and Prevention, Maputo, Mozambique; 6 Clinton Health Access Initiative, Boston, Massachusetts, United States of America; 7 President’s Malaria Initiative, United States Agency for International Development, Maputo, Mozambique; 8 Division of Foodborne, Waterborne, and Environmental Diseases, Centers for Disease Control and Prevention, Atlanta, United States of America; University of Buea, CAMEROON

## Abstract

**Background:**

Universal coverage with long-lasting insecticidal nets (LLINs) is a primary control strategy against *Plasmodium falciparum* malaria. However, its impact on the three other main species of human malaria and lymphatic filariasis (LF), which share the same vectors in many co-endemic areas, is not as well characterized. The recent development of multiplex antibody detection provides the opportunity for simultaneous evaluation of the impact of control measures on the burden of multiple diseases.

**Methodology/Principal findings:**

Two cross-sectional household surveys at baseline and one year after a LLIN distribution campaign were implemented in Mecubúri and Nacala-a-Velha Districts in Nampula Province, Mozambique. Both districts were known to be endemic for LF; both received mass drug administration (MDA) with antifilarial drugs during the evaluation period. Access to and use of LLINs was recorded, and household members were tested with *P*. *falciparum* rapid diagnostic tests (RDTs). Dried blood spots were collected and analyzed for presence of antibodies to three *P*. *falciparum* antigens, *P*. *vivax* MSP-1_19_, *P*. *ovale* MSP-1_19_, *P*. *malariae* MSP-1_19_, and three LF antigens. Seroconversion rates were calculated and the association between LLIN use and post-campaign seropositivity was estimated using multivariate regression. The campaign covered 68% (95% CI: 58–77) of the population in Nacala-a-Velha and 46% (37–56) in Mecubúri. There was no statistically significant change in *P*. *falciparum* RDT positivity between the two surveys. Population seropositivity at baseline ranged from 31–81% for the *P*. *falciparum* antigens, 3–4% for *P*. *vivax* MSP-1_19_, 41–43% for *P*. *ovale* MSP-1_19_, 46–56% for *P*. *malariae* MSP-1_19_, and 37–76% for the LF antigens. The seroconversion rate to the LF Bm33 antigen decreased significantly in both districts. The seroconversion rate to *P*. *malariae* MSP-1_19_ and the LF Wb123 and Bm14 antigens each decreased significantly in one of the two districts. Community LLIN use was associated with a decreased risk of *P*. *falciparum* RDT positivity, *P*. *falciparum* LSA-1 seropositivity, and *P*. *malariae* MSP-1_19_ seropositivity, but not LF antigen seropositivity.

**Conclusions/Significance:**

The study area noted significant declines in LF seropositivity, but these were not associated with LLIN use. The MDA could have masked any impact of the LLINs on population LF seropositivity. The LLIN campaign did not reach adequately high coverage to decrease *P*. *falciparum* RDT positivity, the most common measure of *P*. *falciparum* burden. However, the significant decreases in the seroconversion rate to the *P*. *malariae* antigen, coupled with an association between community LLIN use and individual-level decreases in seropositivity to *P*. *falciparum* and *P*. *malariae* antigens show evidence of impact of the LLIN campaign and highlight the utility of using multiantigenic serological approaches for measuring intervention impact.

## Author summary

*Plasmodium falciparum* malaria is the principal cause of illness and death in Mozambique. However, the same mosquitoes that transmit *P*. *falciparum* parasites also transmit three other species of malaria (*P*. *malariae*, *P*. *ovale*, and *P*. *vivax*) and the worm that causes lymphatic filariasis. To date, we do not know how much transmission of the three other species of malaria occurs. We also do not know if control interventions such as the distribution of bed nets reduce the transmission of lymphatic filariasis and non-falciparum malaria. To address this question, we sampled community members immediately following and one year after a bed net distribution campaign in Mozambique. We analyzed their blood for the presence of antibodies to four species of malaria and lymphatic filariasis. We found that a substantial proportion of individuals had antibodies to *P*. *falciparum*, *P*. *malariae*, *P*. *ovale*, and the worms causing lymphatic filariasis. We found much lower rates of seropositivity to *P*. *vivax*. Individuals reporting using bed nets had a lower risk of testing positive for *P*. *falciparum* and *P*. *malariae* antibodies. The proportion of the population with access to and using bed nets was too low to cause a population-wide decrease in malaria transmission. There was a significant decline in lymphatic filariasis seropositivity between the two surveys, but we could not attribute it to the bed net distribution campaign. Measuring antibody levels for multiple diseases simultaneously has utility in assessing intervention impact.

## Introduction

Northern Mozambique has one of the highest rates of *Plasmodium falciparum* transmission and disease burden in the world [1]. As in the rest of Mozambique, infection with *P*. *falciparum* malaria parasites is among the principal causes of outpatient visits, hospitalizations, and deaths. Malaria transmission occurs year-round and *P*. *falciparum* prevalence in children under 5 was measured to reach up to 65% in the central and northern provinces in 2015 [2].

Although *P*. *falciparum* parasites are the most deadly and common agent transmitted by *Anopheles* vectors in Mozambique, anopheline mosquitoes are also responsible for transmission of three other species of human malaria–*P*. *malariae*, *P*. *ovale*, and *P*. *vivax*–as well as the lymphatic filariasis (LF) parasite *Wuchereria bancrofti*. In contrast to *P*. *falciparum*, the distribution and burden of non-falciparum malaria and LF in Mozambique, as in most of sub-Saharan Africa, has not been well characterized. Reasons for this include poor diagnostic capability, less severe manifestations of disease, and less attention and funding from ministries of health and international donors. In general, *P*. *vivax* has historically been thought to be largely absent from sub-Saharan Africa due to the lack of the Duffy coat receptor in populations originating in West Africa [3]. Although *P*. *ovale* and *P*. *malariae* are thought to be in circulation in Mozambique, estimating their incidence of infection has been difficult, particularly since they are typically present at low parasite densities and are difficult to detect through slide microscopy, especially in the presence of a concomitant *P*. *falciparum* infection. Lymphatic filariasis has been mapped to be most prevalent in northern and central Mozambique [4], but the highly focal nature of LF transmission and the delay between infection and development of disease makes surveillance of this major cause of disability difficult.

Currently, the most effective strategies for reducing the burden of *P*. *falciparum* infection focus on vector control [5]. In Mozambique, *Anopheles* mosquitoes are targeted by indoor residual spraying with insecticides and the distribution and use of long-lasting insecticidal nets (LLINs), which, in addition to protecting the user with a physical barrier, in practice also function as human-baited insecticidal mosquito traps and can significantly reduce mosquito populations [6]. The Mozambican National Malaria Control Program (NMCP) adopted a strategy of universal coverage with LLINs throughout the country in 2010–2011, aiming to cover each sleeping space with an LLIN. Implementation began with a series of sub-provincial mass distribution campaigns, some of which were implemented by the Ministry of Health and local authorities, and some by non-governmental partner organizations. Although primarily used to prevent *P*. *falciparum* infections, LLINs are known to reduce transmission of *P*. *vivax* [6] and have been postulated to also reduce transmission of *P*. *ovale* and *P*. *malariae* [7] and the LF parasites through their effect on the common vector [8]. Although there is some evidence of the impact of LLINs on LF transmission [8, 9], nets have not been widely adopted as a primary intervention against LF, with current strategies largely limited to mass drug administrations (MDAs) of the antifilarial drugs ivermectin or diethylcarbamazine in combination with albendazole [10].

As part of its monitoring and evaluation program, the Mozambican NMCP periodically evaluates LLIN distribution campaigns. The objectives are to both monitor the operational performance of the campaigns, as assessed through coverage and usage indicators, as well as to measure the impact of the campaigns on malaria prevalence and estimates of transmission. The latter is particularly important amid the rise of insecticide resistance and the potential for diminishing effectiveness of LLINs in controlling malaria transmission. In 2013, the Mozambican NMCP chose to evaluate a LLIN distribution campaign in the northern province of Nampula. Besides measuring coverage and falciparum malaria prevalence, additional components were added to the evaluation to measure the prevalences and assess the impact of the campaign on non-falciparum Plasmodium species. A further component was included to assess the additional impact of the LLIN campaign on LF; this was complex as both districts received MDAs for LF during the evaluation period. These additional objectives were made possible by the recent development of multiplex serology methods that allow detection of antibodies to multiple antigens simultaneously [11]. It was hypothesized that this laboratory technique could detect changes in the levels of antibodies to various malaria and LF antigens following the campaign, indicating changes in exposure to, and hence transmission of, malaria and LF parasites as a result of the LLIN campaign.

## Methods

### Study design

Two consecutive cross-sectional household surveys were carried out one year apart, with the first, baseline survey occurring two weeks following the mass LLIN distribution campaign in 2013. Achieved coverage with LLINs was assessed during the first survey, LLIN usage was assessed during the follow-up survey one year later in 2014, and impact of the campaign was assessed by comparison of biological markers of infection from the first and second surveys.

### Study area and population

A LLIN distribution campaign encompassing six districts of the northern province of Nampula was implemented in the second half of 2013. Of the six districts, two were purposefully chosen to be included in the survey: the coastal district of Nacala-a-Velha, and Mecubúri District in the interior (S1 Fig). Both districts are predominantly rural. Nacala-a-Velha is in close proximity to the port city of Nacala-Porto, and Mecubúri, although close to the provincial capital of Nampula, is particularly difficult to access. Both districts were classified as endemic for LF as of 2013, and began undergoing annual MDAs of albendazole and ivermectin starting 2012 in Nacala-a-Velha and starting 2013 in Mecubúri. Administrative coverage for the MDAs in Nacala-a-Velha and Mecubúri was 70% and 114%, respectively, in 2013 and 76% and 82% in 2014.

For the survey, twenty enumeration areas (survey clusters) in each district were chosen randomly with probability proportional to size from the full list of census enumeration areas for each district. Four clusters in Mecubúri were inaccessible and were substituted with four randomly chosen replacement clusters. Immediately prior to the start of the survey, trained enumerators visited each selected cluster and compiled a full list of households, recording the name of head of household and the latitude/longitude coordinates of the household. For each cluster, the list of households was randomly sorted, and in the first survey in 2013, survey teams visited households according to the order of the list, continuing until 16 households were visited per cluster. In the second survey in 2014, survey teams revisited households from 2013, matching households based on the name of head of household and coordinates, and no replacement of households was allowed. In each household, all household members present were invited to participate in the survey. The target sample size was 1,320 individuals and 367 households per district, designed to provide 80% power to detect a 10% change between the two surveys in the proportion of the population testing positive for *P*. *falciparum* parasites, assuming a baseline prevalence of 43% and a design effect of 3.

### Data collection

Trained teams, each composed of a national-level supervisor and a district surveyor, visited the selected households and, after obtaining consent, administered a household questionnaire. Surveyors collected socioeconomic data, including occupation and education of the head of household and household ownership of goods; generated a roster of household members; enumerated all sleeping spaces and recorded who slept in which sleeping space; visually inspected each sleeping space and recorded the presence and location (hanging or stored) of the bed net designated for that sleeping space, denoting whether or not the bed net bore the marking specific to the distribution campaign; and asked the interviewee about how often on average each bed net was used during the wet and dry seasons. In both years, each household member, regardless of age, present during the visit and providing written consent was administered a *P*. *falciparum* HRP2-specific rapid diagnostic test (RDT) (SD Bioline, Yongin, Republic of Korea), and had up to six 10 mcL spots of capillary blood collected on filter paper (TropBio, Cellabs, Sydney, Australia). Individuals testing positive for malaria were treated by survey teams in accordance with national treatment guidelines [12]. In 2014, the same questionnaires and procedures were followed as in 2013, with an additional module where household members were cross-linked to those in the first round based on name and age.

The surveys were carried out in September 2013 and October 2014 in Nacala-a-Velha and December 2013 and November 2014 in Mecubúri, with data collection lasting three weeks for each survey in each district.

### Laboratory analysis

After collection in the field, the filter paper was dried overnight, placed into individual plastic bags with desiccant sachets, and then refrigerated prior to shipment to central laboratories in Maputo and later to CDC laboratories in Atlanta. Blood spots were eluted overnight at 4°C at a serum dilution of 1:40 (assuming 50% hematocrit) and further diluted in casein-containing dilution buffer as previously described for a final dilution of 1:400 of serum [13, 14].

A multiplex bead platform [11] was used to measure immunoglobulin G (IgG) antibody response to ten antigens: six malaria, three LF, and one control antigen (*Strongyloides stercoralis*) (S1 Table). The 19-kDa subunit of the merozoite surface protein 1 (MSP-1_19_) from each of the four main human malaria species was cloned and expressed as recombinant *Schistosoma japonicum* glutathione-*S*-transferase (GST) fusion proteins [14–16]. A *P*. *vivax* MSP-1_19_ expression clone that included the carboxy-terminal hydrophobic tail sequence was used [16]. A (NANP)_5_ peptide corresponding to the carboxy-terminus of the *P*. *falciparum* circumsporozoite protein (CSP) was cross-linked to GST and then coupled to a SeroMap bead as previously described [14]. The Pl1043 epitope from *P*. *falciparum* Liver Stage Antigen 1 (LSA-1) [17] was synthesized and coupled to beads at a concentration of 60ug/mL at pH 5.0. The *Strongyloides stercoralis* NIE antigen-GST fusion protein, GST fusion partner with no inserted sequence, and the *Brugia malayi* Bm14- and Bm33-GST fusion proteins were cloned and expressed as previously described [18–21]. *W*. *bancrofti* Wb123 antigen expressed as a GST fusion protein was a gift of T. Nutman (NIH, Bethesda, MD). Although only *W*. *bancrofti* occurs in Africa, the serological test for the *B*. *malayi* antigens cross-reacts with *W*. *bancrofti*.

With the exception of the *P*. *vivax* GST/MSP-1_19_, all other antigens were coupled to SeroMap beads (Luminex Corp., Austin, TX) using the buffers and protein amounts previously described [16]. The *P*. *vivax* antigen was coupled to a BioPlex COOH bead (BioRad, Hercules, CA) using the protein amount and buffer previously specified [16]. Total IgG multiplex bead assays were performed using the biotin-streptavidin system previously described [19, 20]. Assays included beads coated with the 10 proteins described above and an additional 31 antigen-coated beads representing viral, bacterial, and parasitic diseases. Each assay plate included a buffer-only blank and 6 control sera to ensure consistent assay performance throughout the study. Assays were run in duplicate, and results were reported as the average of the two median fluorescent intensity values *minus* the buffer-only blank value (MFI-bg). Samples (N = 8) that had discordant result between the two runs (coefficient of variation >15%) for >4 positive antigen responses were repeated.

For malaria and LF antigens, cutoff values were determined using a panel of 81 presumed negative sera from adult US citizens who had no history of foreign travel. Values greater than the mean *plus* 3 standard deviations of these negative control values were considered positive. For the *S*. *stercoralis* NIE assay, a cutoff value determined by a receiver-operator characteristic curve analysis using a different lot of coupled beads was translated to the current study bead set using a 2-fold serial dilution curve of a strong positive control sample as an inter-assay standard.

A subset of samples (20%) did not have full blood spots available; for these, partly filled blood spots were analyzed. Comparison of average MFI-bg values from full and partial blood spots revealed an average difference of <10%; thus, all samples were included in the final statistical analysis.

### Statistical analysis

Demographic characteristics for the heads of household were tabulated, and a socioeconomic status (SES) index was constructed and calculated for each household using a previously described methodology [22]. Households were divided into equal quintiles based on the SES index score. Key ownership, access, and usage indicators [23] were calculated separately for each district. The proportion of the population testing positive for *P*. *falciparum* infection by RDT was calculated for each district and year, stratifying by age. Estimation of coverage indicators and malaria positivity was adjusted taking into account the complex sample design using the R **survey** package [24]. Data were weighted by the inverse of the probability of selection, calculated as the product of the probability of selection for the cluster, the household, and, where applicable, the individual.

For each of the antigens the mean seropositivity, defined as the percent of individuals with MFI-bg above the predetermined threshold, was calculated for the total population and also stratifying by ten age categories. A reversible catalytic model was fit to the seropositivity by age data for each antigen, and the estimates for the serological conversion rate (SCR) and serological reversion rate (SRR) per year were directly calculated from the likelihood model [25]. The SRR was assumed to be constant for both districts and both years, but the SCR was separately calculated for each district and each year of the survey. For each antigen, the average of the population log MFI-bg value was calculated for each district and year, and the 95% confidence intervals were calculated assuming a normal distribution of the log MFI-bg values. The *Strongyloides* NIE antigen was included as a control antigen to aid in discriminating between the effects of the LLIN and MDA campaigns, as *Strongyloides* transmission was presumed to be unaffected by the LLIN campaigns but sensitive to the MDA campaigns.

Poisson regression, which allows direct estimation of the relative risk, [26] was used to model the association between individual- and cluster-level LLIN use and ten binary biological outcomes in individuals sampled in the second survey: RDT positivity and seropositivity for the six malaria and three LF antigens. Each model was fit adjusting for age, sex, and household SES. Use of LLIN was quantified on a 0 to 1 scale by a principal components analysis of data on LLIN location and reported use during the wet and dry season, as previously described [23]. The individual and community effects were jointly estimated by normalizing the individual-level variable by subtracting the average cluster-level value from the individual-level variable [27].

All statistical analyses were performed in R version 3.3.2 (R Foundation for Statistical Computing, Vienna, Austria).

### Ethical considerations

The study was approved by the National Bioethics Committee in Mozambique. Adult participants provided written consent prior to enrollment in the study, and also provided written consent on behalf of child participants. CDC investigators provided technical assistance and were not considered to be engaged in the research.

## Results

A total of 282 households in Nacala-a-Velha and 300 households in Mecubúri were visited in the first survey in 2013 (Table 1). The total number of people living in these households was 1,172 in Nacala-a-Velha and 1,443 in Mecubúri, and of these, 539 (46%) household members in Nacala-a-Velha and 598 (41%) in Mecubúri were present and consented to have blood drawn during the survey. In the follow up survey in 2014, 81% (228/282) of the households in Nacala-a-Velha and 72% (217/300) in Mecubúri were revisited; a total of 578 household members in Nacala-a-Velha and 704 in Mecubúri were sampled in the second survey.

**Table 1:** Demographic data on study population of household surveys in Nacala-a-Velha and Mecubúri Districts, Mozambique, 2013–2014.

	Nacala-a-Velha		Mecubúri	
	2013	2014	2013	2014
Households visited	282	228	300	217
Total household members	1172	908	1443	971
Total household members sampled	539	578	598	704
<5 years	124 (23%)	123 (21%)	131 (22%)	146 (21%)
5–14 years	142 (26%)	163 (28%)	142 (24%)	219 (31%)
>14 years	273 (51%)	292 (51%)	325 (54%)	339 (48%)
Female	299 (55%)	310 (54%)	326 (55%)	384 (55%)
Household members sampled both years	275		302	
<5 years	45 (16%)		53 (18%)	
5–14 years	87 (32%)		89 (29%)	
>14 years	143 (52%)		160 (53%)	
Female	159 (58%)		175 (58%)	

The coverage attained by the LLIN distribution campaign was low (Table 2). The campaign reached 80% (95% CI: 72–86) of households in Nacala-a-Velha and 54% (44–65) in Mecubúri, but only 58% (48–67) of households in Nacala-a-Velha and 36% (29–43) in Mecubúri received at least one LLIN per sleeping space. The proportion of the population sleeping in spaces with an available LLIN was 68% (58–77) in Nacala-a-Velha and 46% (37–56) in Mecubúri. Usage of any LLIN in the year following the distribution campaign was also low, with only 40% (27–55) of the population in Nacala-a-Velha and 23% (17–30) in Mecubúri reporting having used LLINs more than 4 times per week during the wet season, falling to 21% (14–30) and 17% (13–22), respectively, in the dry season.

**Table 2:** Coverage with LLINs immediately following a mass LLIN distribution campaign in 2013 in Nacala-a-Velha and Mecubúri Districts, Mozambique.

	Nacala-a-Velha	Mecubúri
	% (95% CI)	% (95% CI)
Ownership^1^		
Households receiving at least one campaign LLIN	80 (72–86)	54 (44–65)
Access^1^		
Households receiving at least one LLIN per sleeping space	58 (48–67)	36 (29–43)
Sleeping spaces covered by campaign LLIN	66 (58–74)	43 (35–52)
People with access to campaign LLIN	68 (58–77)	46 (37–56)
Usage^2^		
Sleeping spaces with a hung campaign LLIN	30 (19–43)	17 (11–23)
Sleeping spaces with a campaign LLIN reported to be used ≥4 times a week during dry season^3^	21 (14–30)	17 (13–22)
Sleeping spaces with a campaign LLIN reported to be used ≥4 times a week during wet season^3^	40 (27–55)	23 (17–30)

^1^Assessed immediately following campaign

^2^Assessed one year after campaign

^3^In preceding year, self-reported

LLIN: long-lasting insecticidal net

There was no statistically significant change in *P*. *falciparum* RDT positivity from 2013 in 2014 in either district with overlapping 95% confidence intervals, although the point estimates for RDT positivity were higher in 2014 versus 2013 (Table 3). One year after the campaign, RDT positivity in the key <5 year age group was 61% (95% CI: 44–76) in Nacala-a-Velha and 87% (76–94) in Mecubúri. Overall, RDT positivity was significantly higher in Mecubúri than in Nacala-a-Velha.

**Table 3:** Prevalence of *P*. *falciparum* infection as assessed by RDT immediately following and one year after an LLIN distribution campaign in Nacala-a-Velha and Mecubúri Districts, Mozambique.

	% RDT+ (95% Confidence Interval)	
	2013	2014
Nacala-a-Velha		
All ages	44 (33–56)	48 (35–61)
<5 years	52 (36–67)	61 (44–76)
5–14 years	63 (34–87)	67 (38–89)
>14 years	30 (22–40)	30 (22–39)
Mecubúri		
All ages	65 (56–74)	70 (64–76)
<5 years	67 (52–80)	87 (76–94)
5–14 years	89 (77–96)	93 (87–97)
>14 years	54 (43–65)	49 (41–56)

LLIN: long-lasting insecticidal net

RDT: rapid diagnostic test

**Fig 1:**
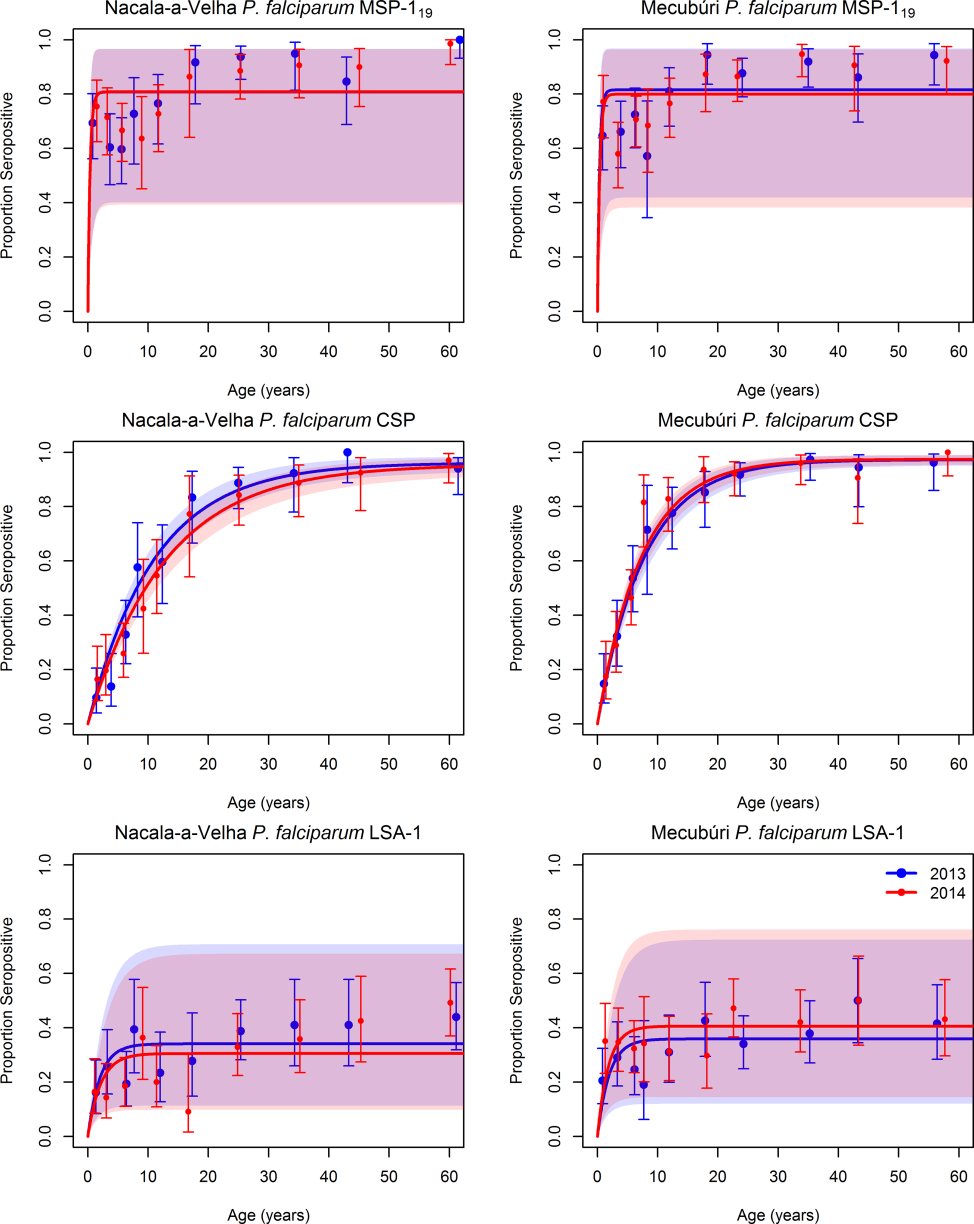
Seropositivity by age for antibodies to three *P*. *falciparum* antigens in community members sampled during household surveys in Nacala-a-Velha and Mecubúri Districts, Northern Mozambique, 2013–2014. Points represent estimates and 95% confidence intervals for seropositivity for each age category, curves represent the fit of a catalytic conversion model, and shaded areas represent the 95% confidence intervals of model fit: blue for 2013, red for 2014, and purple for the overlap.

The serological data confirm high *P*. *falciparum* transmission in both districts. Virtually all sampled individuals were positive for *P*. *falciparum* MSP-1_19_ antibodies, with very high antibody responses (S2 Fig) even in infants, indicating that individuals’ first *P*. *falciparum* infection likely occurs early in infancy. Seropositivity for *P*. *falciparum* CSP also eventually reached saturation, with close to 100% of the older age categories testing positive, but the slope of the seropositivity by age curve was more gradual (Fig 1). In contrast to the other two *P*. *falciparum* antigens, seropositivity to *P*. *falciparum* LSA-1 in general did not surpass 40% for any age group.

For both *P*. *falciparum* CSP and *P*. *falciparum* LSA-1 antigens, the SCR was higher in Mecubúri than Nacala-a-Velha, consistent with the difference in RDT positivity by district. Generally, there was no statistically significant difference in SCR between the two years for these antigens (t-test p-values ranging from 0.07 to 0.35) (Table 4). The only difference approaching statistical significance was a slightly lower SCR for *P*. *falciparum* CSP in 2014 versus 2013 in Nacala-a-Velha, which fell by 15% (t-test p-value 0.07).

**Table 4:** Change in serological indicators of malaria and lymphatic filariasis exposure immediately following and one year after an LLIN distribution campaign in Nacala-a-Velha and Mecubúri Districts, Mozambique.

	Serological conversion rate				log Median Fluorescent Intensity (paired analysis)				Seroposivity			
	2013	2014	% change	p-value	2013	2014	% change	p-value	2013	2014	% change	p-value
Nacala-a-Velha												** **
*P*. *falciparum* MSP-1_19_ antigen	2.263	2.271	0%	0.50	3.688	3.782	3%	0.208	80%	80%	0%	0.99
*P*. *falciparum* CSP antigen	0.087	0.074	-15%	0.07	3.493	3.494	0%	0.986	61%	58%	-5%	0.33
*P*. *falciparum* LSA-1 antigen	0.17	0.144	-15%	0.35	2.198	2.227	1%	0.617	31%	28%	-10%	0.27
*P*. *vivax* MSP-1_19_ antigen	0.002	0.001	-38%	0.20	2.279	2.238	-2%	0.24	3%	2%	-37%	0.37
*P*. *ovale* MSP-1_19_ antigen	0.118	0.092	-22%	0.10	2.076	2.1	1%	0.632	41%	35%	-14%	0.06
*P*. *malariae* MSP-1_19_ antigen	0.068	0.051	-24%	**0.03**	2.689	2.641	-2%	0.556	46%	40%	-14%	**0.04**
*W*. *bancrofti* Wb123 antigen	0.074	0.054	-27%	**0.01**	2.502	2.402	-4%	0.16	50%	43%	-15%	**0.02**
*B*. *malayi* Bm14 antigen	0.031	0.026	-17%	0.08	2.282	2.191	-4%	0.323	37%	32%	-12%	0.16
*B*. *malayi* Bm33 antigen	0.169	0.131	-22%	**0.03**	3.087	3.002	-3%	0.066	67%	61%	-10%	**0.04**
*Strongyloides* NIE antigen (control)	0.125	0.114	-8%	0.26	3.265	3.198	-2%	0.278	58%	56%	-4%	0.53
Mecubúri												** **
*P*. *falciparum* MSP-1_19_ antigen	2.382	2.151	-10%	0.42	3.764	3.741	-1%	0.729	81%	80%	-2%	0.61
*P*. *falciparum* CSP antigen	0.125	0.133	7%	0.27	3.677	3.709	1%	0.631	71%	71%	0%	0.99
*P*. *falciparum* LSA-1 antigen	0.184	0.224	22%	0.32	2.342	2.324	-1%	0.773	33%	38%	15%	0.09
*P*. *vivax* MSP-1_19_ antigen	0.003	0.001	-53%	0.07	2.331	2.211	-5%	**9E-04**	4%	2%	-54%	**0.04**
*P*. *ovale* MSP-1_19_ antigen	0.125	0.121	-3%	0.43	2.153	2.114	-2%	0.412	43%	42%	-2%	0.78
*P*. *malariae* MSP-1_19_ antigen	0.095	0.094	-1%	0.46	2.919	2.912	0%	0.918	56%	54%	-3%	0.59
*W*. *bancrofti* Wb123 antigen	0.048	0.039	-19%	0.05	2.321	2.203	-5%	**0.043**	41%	35%	-15%	**0.03**
*B*. *malayi* Bm14 antigen	0.044	0.03	-31%	**8.E-04**	2.438	2.289	-6%	0.12	45%	34%	-23%	**2.E-04**
*B*. *malayi* Bm33 antigen	0.246	0.101	-59%	**2.E-09**	3.146	2.885	-8%	**9E-09**	76%	55%	-27%	**1.E-13**
*Strongyloides* NIE antigen (control)	0.163	0.105	-35%	**9.E-04**	3.43	3.242	-5%	**0.005**	65%	54%	-17%	**8.E-05**

P-values for differences in serological conversion rate were derived with a normal distribution test, log Median Fluorescent Intensity with a two-sample t-test, and seropositivity with Pearson's chi-squared test

**Fig 2:**
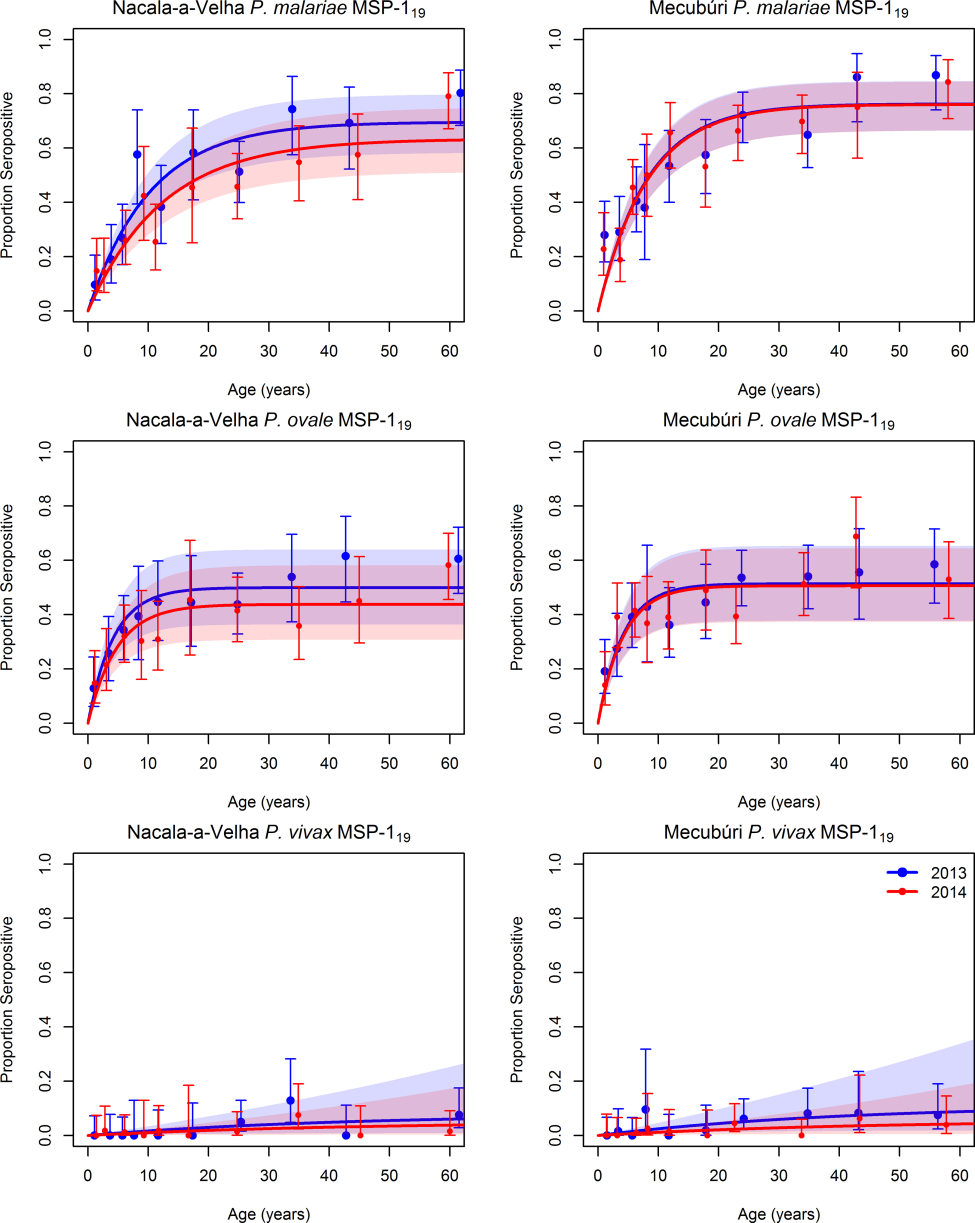
Seropositivity by age for antibodies to *P*. *ovale*, *P*. *malaria*, *and P*. *vivax* MSP-1_19_ antigens in community members sampled during household surveys in Nacala-a-Velha and Mecubúri Districts, Northern Mozambique, 2013–2014. Points represent estimates and 95% confidence intervals for seropositivity for each age category, curves represent the fit of a catalytic conversion model, and shaded areas represent the 95% confidence intervals of model fit: blue for 2013, red for 2014, and purple for the overlap.

Robust antibody responses to the three other *Plasmodium* species were detected in both young and old individuals in both districts (Fig 2, S3 Fig), an indication of ongoing transmission of all three other species. After *P*. *falciparum*, the species registering the highest seropositivity was *P*. *malariae*, with 46% (95%CI: 42–50) of the population in 2013 in Nacala-a-Velha and 56% (52–60) in Mecubúri seropositive for antibodies against *P*. *malariae* MSP-1_19_, with a corresponding SCR of 0.068 (0.054–0.081) in Nacala-a-Velha and 0.095 (0.077–0.11) in Mecubúri. Similar levels of exposure were observed for *P*. *ovale*, with the proportion of the population with antibodies to *P*. *ovale* MSP-1_19_ in 2013 ranging from 41% (37–45) in Nacala-a-Velha to 43% (39–47) in Mecubúri, and an SCR estimated to be 0.12 (0.087–0.15) in Nacala-a-Velha and 0.13 (0.093–0.16) in Mecubúri. Much lower levels of antibody positivity to *P*. *vivax* were observed, with only 2.7% (1.5–4.5) of the population in Nacala-a-Velha and 3.9% (2.5–5.9) in Mecubúri with detectable antibodies to *P*. *vivax* MSP-1_19_. There was a statistically significant difference in SCR for *P*. *malariae* between the two surveys in Nacala-a-Velha, with the estimate for SCR for 2014 24% lower than in 2013 (t-test p-value 0.03). There was a similar reduction in the SCR for *P*. *ovale* in Nacala-a-Velha, with a 22% reduction, but this was not statistically significant (t-test p-value 0.096). The SCRs for *P*. *malariae* and *P*. *ovale* in Mecubúri did not show a similar reduction, falling by only 1% and 3%, respectively, with neither antigen showing a statistically significant difference in SCR between the two surveys (t-test p-values ranging from 0.43 to 0.46).

**Fig 3:**
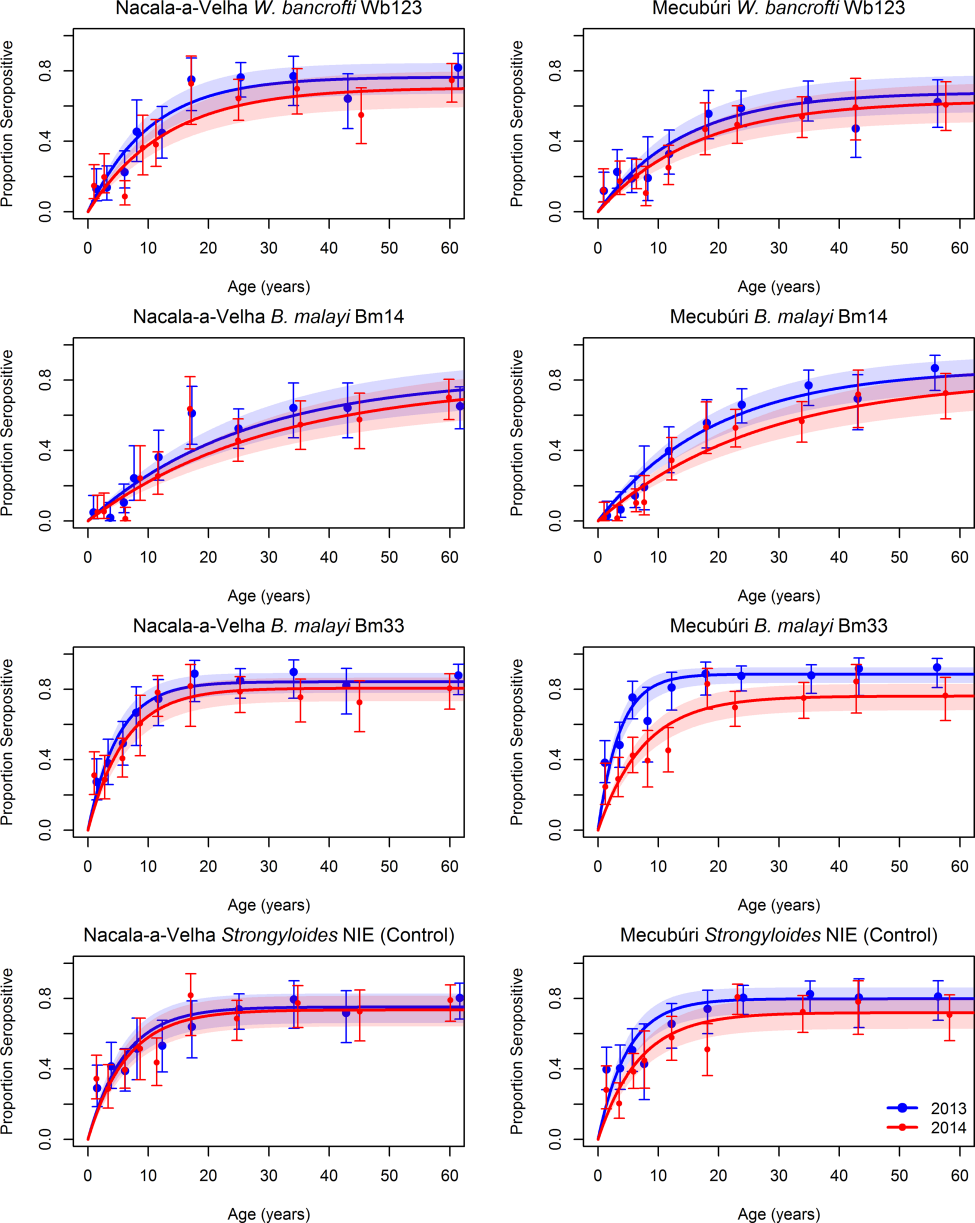
Seropositivity by age for antibodies to three lymphatic filariasis antigens and the *Strongyloides* NIE (control) antigen in community members sampled during household surveys in Nacala-a-Velha and Mecubúri Districts, Northern Mozambique, 2013–2014. Points represent estimates and 95% confidence intervals for seropositivity for each age category, curves represent the fit of a catalytic conversion model, and shaded areas represent the 95% confidence intervals of model fit: blue for 2013, red for 2014, and purple for the overlap.

The absolute levels of antibody response and seropositivity by age curves for the three LF antigens were consistent with the geographic distraction of LF (Fig 3, S4 Fig). The highest rates of seropositivity at baseline were to the Bm33 antigen, with 67% (95%CI: 56–65) of the population in 2013 in Nacala-a-Velha and 76% (72–79) in Mecubúri seropositive, compared to 37% (33–41) in Nacala-a-Velha and 45% (41–49) in Mecubúri seropositive for antibodies against Bm14, and 50% (46–54) in Nacala-a-Velha and 41% (37–45) in Mecubúri seropositive for antibodies against Wb123 (Table 4). There were significant reductions in SCR for the LF antigens between the two surveys in Nacala-a-Velha, with the SCR for Wb123 declining by 27% (chi-square test p-value 0.011), the SCR for Bm14 declining by 17% (chi-square test p-value 0.084), and the SCR for Bm33 declining by 22% (chi-square test p-value 0.026). In Mecubúri, the SCR fell by 19% for Wb123 (t-test p-value 0.052), 31% for Bm14 (t-test p-value <0.001), and 59% for Bm33 (t-test p-value <0.001).

A substantial proportion of the population in Nacala-a-Velha (58%, 95%CI: 53–62) and Mecubúri (65%, 95%CI: 61–69) had antibodies against the control *Strongyloides* NIE antigen in 2013. In Nacala-a-Velha, there was no statistically significant difference in SCR (t-test p-value 0.26) or seropositivity (chi-square p-value 0.53) to the NIE antigen between the two surveys, whereas Mecubúri witnessed statistically significant declines of 35% for the NIE SCR (t-test p-value <0.01) and 17% for NIE seropositivity (chi-square p-value <0.01) from 2013 to 2014.

The achieved sample size was lower than the target sample size, due to a lower than expected number of people tested per household (2.4 vs 3.6). Although there was no detected change in overall *P*. *falciparum* RDT positivity from 2013 to 2014, both individual- and cluster-level LLIN use was associated with lower risk for *P*. *falciparum* RDT positivity in the second year after adjusting for age, sex, and SES (Table 5). The relative risk for testing RDT positive in LLIN users compared to non-users was 0.81 (95%CI: 0.64–1.0). Moreover, the relative risk of RDT positivity for the individuals living in clusters where everyone would be sleeping under an LLIN was estimated to be 0.43 (0.24–0.77) compared to individuals living in clusters with no LLIN use, demonstrating the indirect effects of LLINs, independent of individual use of LLINs. Cluster-level LLIN use was also associated with lower risk for seropositivity to *P*. *falciparum* LSA-1 and *P*. *malariae* MSP-1_19_ antigens. In contrast, no significant protective effect of individual- or community-level LLIN use on LF, *P*. *vivax* MSP-1_19_, and *P*. *ovale* MSP-1_19_ antibody positivity was observed. Generally, RDT positivity and seropositivity to all antigens decreased with increasing SES. *P*. *falciparum* RDT positivity was negatively associated with age, in contrast to antibody seropositivity, which increased with age for all antigens.

**Table 5:** Relative risk for malaria and lymphatic filariasis outcomes one year after an LLIN distribution campaign in Nacala-a-Velha and Mecubúri Districts, Mozambique, as a function of demographic, socioeconomic, and bed net usage factors.

		Antibody seropositivity in 2014								
	Malaria RDT+ in 2014	*P*. *falciparum* MSP-1_19_	*P*. *falciparum* CSP	*P*. *falciparum* LSA-1	*P*. *vivax* MSP-1_19_	*P*. *ovale* MSP-1_19_	*P*. *malariae* MSP-1_19_	*W*. *bancrofti* Wb123	*B*. *malayi* Bm14	*B*. *malayi* Bm33
Age										
<5 yrs	Ref	Ref	Ref	Ref	Ref	Ref	Ref	Ref	Ref	Ref
5–10 yrs	1.1 (0.92–1.3)	0.99 (0.8–1.2)	2.1 (1.5–3)	1.1 (0.81–1.6)	2.8 (0.29–27)	1.6 (1.2–2.3)	2.2 (1.6–3.2)	1.0 (0.68–1.6)	2.7 (1.2–6.1)	1.5 (1.1–2.1)
10–15 yrs	1.1 (0.85–1.3)	1.1 (0.84–1.4)	3.4 (2.4–4.8)	1 (0.68–1.6)	4.3 (0.39–48)	1.6 (1.1–2.4)	2.7 (1.8–4)	1.9 (1.2–2.9)	8.9 (4.1–19)	2.1 (1.5–2.9)
15–20 yrs	0.82 (0.61–1.1)	1.2 (0.91–1.6)	4.2 (2.9–6.1)	0.9 (0.52–1.6)	1e-06 (0-Inf)	2.0 (1.3–3.1)	2.8 (1.8–4.4)	3.6 (2.3–5.6)	18 (8.4–39)	3.0 (2.1–4.3)
20–30 yrs	0.56 (0.43–0.73)	1.2 (0.99–1.6)	4.2 (3.1–5.8)	1.6 (1.1–2.3)	7.9 (0.91–67)	1.7 (1.2–2.5)	3.3 (2.3–4.7)	3.5 (2.4–5.2)	15 (7.5–32)	2.6 (1.9–3.5)
30–40 yrs	0.46 (0.34–0.63)	1.3 (1–1.7)	4.4 (3.2–6.2)	1.5 (1.1–2.2)	7.2 (0.8–65)	1.9 (1.3–2.8)	3.6 (2.5–5.2)	3.8 (2.6–5.6)	17 (8.4–36)	2.7 (2–3.6)
>40 yrs	0.31 (0.23–0.42)	1.3 (1.1–1.7)	4.5 (3.3–6.2)	1.8 (1.3–2.5)	6.8 (0.79–59)	2.5 (1.8–3.4)	4.2 (3–5.9)	3.9 (2.7–5.6)	20 (9.7–40)	2.8 (2.1–3.7)
Sex										
Female	Ref	Ref	Ref	Ref	Ref	Ref	Ref	Ref	Ref	Ref
Male	1.0 (0.9–1.2)	0.87 (0.76–0.99)	0.98 (0.85–1.1)	0.92 (0.75–1.1)	1.4 (0.59–3.5)	0.8 (0.66–0.97)	1.0 (0.84–1.2)	1.2 (1–1.5)	1.3 (1–1.6)	0.95 (0.81–1.1)
SES quintile										
1st (Poorest)	Ref	Ref	Ref	Ref	Ref	Ref	Ref	Ref	Ref	Ref
2nd	0.94 (0.75–1.2)	0.96 (0.77–1.2)	1.1 (0.84–1.4)	1.1 (0.82–1.6)	5.7 (0.63–51)	1.2 (0.89–1.7)	1.1 (0.86–1.5)	0.94 (0.68–1.3)	0.80 (0.57–1.1)	1.0 (0.77–1.3)
3rd	0.90 (0.72–1.1)	0.99 (0.8–1.2)	0.99 (0.78–1.2)	0.94 (0.68–1.3)	3.5 (0.36–34)	1.3 (0.94–1.7)	1 (0.78–1.3)	0.93 (0.7–1.2)	0.78 (0.57–1.1)	0.98 (0.76–1.2)
4th	0.87 (0.7–1.1)	0.94 (0.76–1.2)	0.96 (0.76–1.2)	0.89 (0.64–1.2)	7.9 (0.95–65)	1.1 (0.79–1.5)	0.92 (0.71–1.2)	0.91 (0.68–1.2)	0.77 (0.57–1.1)	0.96 (0.75–1.2)
5th (Richest)	0.80 (0.64–0.99)	0.95 (0.78–1.2)	0.96 (0.77–1.2)	1.0 (0.76–1.4)	4.6 (0.52–41)	0.99 (0.73–1.3)	0.87 (0.66–1.1)	0.8 (0.59–1.1)	0.71 (0.52–0.97)	0.98 (0.77–1.3)
LLIN Use (individual)	0.81 (0.64–1.0)	0.99 (0.82–1.2)	0.98 (0.79–1.2)	1.1 (0.82–1.5)	0.85 (0.23–3.1)	1.0 (0.8–1.4)	0.98 (0.76–1.3)	1.1 (0.83–1.4)	0.97 (0.73–1.3)	1.0 (0.81–1.3)
LLIN Use (community)	0.43 (0.24–0.77)	1.0 (0.6–1.7)	0.71 (0.39–1.3)	0.34 (0.15–0.79)	0.22 (0.0062–8)	0.67 (0.31–1.5)	0.4 (0.2–0.81)	2.0 (0.95–4.4)	0.98 (0.43–2.3)	3.7 (2–6.9)

RDT: rapid diagnostic test; SES: socioeconomic status; LLIN: long-lasting insecticidal net

## Discussion

The high rates of *P*. *falciparum* RDT positivity observed in these surveys, coupled with the high rates and age distribution of antibody levels to *P*. *falciparum* antigens, confirm holoendemic transmission of *P*. *falciparum* in the survey area. The levels of RDT positivity are particularly striking given that the surveys were conducted at the end of the dry season, when transmission would be expected to be lowest. Areas of large malaria burden would benefit most from a mass LLIN campaign. However, the coverage indicators provide evidence that the LLIN campaign evaluated here was far from reaching its intended target of universal coverage. Reported usage, ranging from 17% to 40%, was far removed from the 65% threshold postulated to be necessary in providing a demonstrable community reduction in malaria incidence [28]. Nevertheless, those individuals using LLINs and living in clusters with high overall usage of LLINs did have significantly lower risk for testing positive for *P*. *falciparum* infection by RDT, mirroring results from previous studies of LLIN effectiveness in Mozambique [22] and confirming the continued effectiveness of LLINs as a malaria prevention strategy in Mozambique.

This study highlights the added benefit of simultaneously measuring seropositivity to multiple antigens to estimate *P*. *falciparum* transmission intensity. The results show that not all antigens are consistently informative in this setting. For example, the *P*. *falciparum* MSP-1_19_ antigen, which to date has been one of the standard antigens used to assess population-level *P*. *falciparum* exposure [25, 29] provides little information on changes in *P*. *falciparum* intensity in a setting of such high transmission as northern Mozambique. The transmission intensity is such that virtually all sampled individuals regardless of age had antibodies to MSP-1_19_, evidence that the first *P*. *falciparum* infection likely occurs in early infancy. However, the two other *P*. *falciparum* antigens included in the assay, CSP and LSA-1, generated seroprevalence curves with increasing likelihood of transitioning to seropositive with increasing age. Both antigens are thought to be less immunogenic than MSP-1_19_, and the data presented here suggest that repeated *P*. *falciparum* infections throughout life are needed to generate a consistently high antibody level to each antigen. Due to the slower acquisition of antibodies to these two antigens, the seroprevalence by age data were informative, allowing differentiation between the higher transmission in Mecubúri versus Nacala-a-Velha, an observation also seen in the RDT positivity data. Additionally, *P*. *falciparum* LSA-1 seropositivity was lower in LLIN users, further evidence of the protective effect of LLINs against *P*. *falciparum* infection.

In addition to the two districts being a setting of very high *P*. *falciparum* transmission, the populations in both districts showed substantial serological responses to *P*. *ovale* and *P*. *malariae* antigens. A small but non-zero proportion of the population showed evidence of exposure to *P*. *vivax*, consistent with the results of a 2015 household survey which showed a national 0.2% *P*. *vivax* prevalence in children under 5 years of age [2]. MSP-1_19_ antigen competition studies have not indicated antibody cross-reactivity in most individuals [14], and species-specific MSP-1_19_ antibody responses were common even among patients who had high responses to multiple malaria MSP-1_19_ antigens. Although the MSP-1_19_ antigens from *P*. *vivax* and *P*. *falciparum* share 51% identity at the amino acid level, some of the conserved residues are cysteines and other hydrophobic amino acids that are unlikely to be exposed to the immune response [30]. Bousema et al. used the two MSP-1_19_ antigens in ELISA studies of sera from a population living in a region endemic for both parasites and did not observe any correlation between the *P*. *vivax* and *P*. *falciparum* antibody responses [31]. In a separate study, 79% of women who were positive for antibodies to malaria reacted with the MSP-1_19_ antigen from only one species [16]. Thus, it is unlikely that the observed antibody responses to *P*. *malariae* and *P*. *ovale* antigens can be solely attributed to assay cross-reactivity.

Although subject to many limitations, the SCR at its most basic definition is a measure of incidence, the annual rate at which individuals acquire antibodies to a certain antigen [32]. Taken at face value, the SCRs estimated for *P*. *ovale* MSP-1_19_ and *P*. *malariae* MSP-1_19_ in these two districts in 2013 suggest an annual incidence of *P*. *ovale* infection of 125/1000 in Nacala-a-Velha and 133/1000 in Mecubúri, and an annual incidence of *P*. *malariae* infection of 70/1000 in Nacala-a-Velha and 100/1000 in Mecubúri. This magnitude of incidence would elevate *P*. *ovale* and *P*. *malariae* as major contributors to malaria burden in northern Mozambique.

In contrast to *P*. *falciparum*, there was a statistically significant population-level decrease in *P*. *malariae* seropositivity and a borderline significant decrease in *P*. *ovale* seropositivity from 2013 to 2014 in one of the districts. Since LLIN use was associated with lower post-campaign risk of testing positive for *P*. *malariae* antibodies, there is evidence that the LLIN campaign, at least in Nacala-a-Velha where coverage was higher, might have had an impact on decreased *P*. *malariae* transmission. The fact that there were detectable changes in seroprevalence and distribution of *P*. *ovale* and *P*. *malariae* markers and no change in *P*. *falciparum* might be due to the differences in magnitude of transmission intensity. One hypothesis is that with such high levels of *P*. *falciparum* transmission, there would need to be a much larger decrease in vectorial capacity to result in a detectable change in incidence, whereas non-falciparum transmission might be low enough to be sensitive to smaller changes in vectorial capacity.

In both districts there was evidence of significant declines in LF transmission between 2013 and 2014, as seen by decreases in both the SCR and overall proportion of the population seropositive for the LF antigens. The results are robust as they hold across all three different LF antigens included in the assay. However, attributing this change to the LLIN distribution campaign is hampered by the concurrent MDAs of antiparasitic drugs in both districts. Ivermectin is effective against LF microfilariae, in addition to a postulated killing effect on mosquitos feeding on individuals treated with ivermectin, and albendazole kills adult worms. Together, the two-drug combination could be expected to influence antibody levels through its effect on transmission and worm load. In Mecubúri, the substantial declines in SCR and proportion of the population seropositive for the NIE *Strongyloides* antigen, which should be influenced by the MDAs but not by the LLIN campaign, suggest that there was high enough coverage from the antiparasitic MDAs to reduce LF transmission. In Nacala-a-Velha, however, there was no significant difference in NIE *Strongyloides* SCR and seropositivity between the two surveys, and thus the population-level declines in SCR and seropositivity to LF antigens could be due to the LLIN distribution campaign. Overall, there was no association between individual or community LLIN use and seropositivity to LF antigens. Given the confounding due to the concurrent use of MDAs in the survey districts, this result cannot be interpreted as evidence of no effect, as the effect might have been masked by the antifilarial MDAs.

Several aspects of the study’s design prevent direct inference of a causal relationship between the LLIN distribution campaign and the observed changes in malaria positivity and serological outcomes for malaria and LF. The lack of a control group and the MDA campaigns in the intervening year hamper direct estimation of the impact of the LLIN campaign. In addition, the low coverage and usage resulting from the LLIN campaign and the lower-than-expected sample size limited the ability of the study to assess the impact of LLINs on malaria and LF transmission. Additionally, as IgG against some antigens is known to persist for years following infection [33], more elapsed time may be needed to detect a substantial change in serological metrics following a successful intervention. The extraordinarily high rates of *P*. *falciparum* transmission on the backdrop of low LLIN coverage argue for follow-up campaigns in these two districts, both of which will take part in the nationwide universal coverage campaign in Mozambique launched in 2016. Similar evaluations are recommended to evaluate coverage and usage of future campaigns. Finally, the results presented here provide evidence for the enhanced utility of the multi-antigenic and multi-disease assay for quantifying baseline exposure to the non-falciparum malarias and LF, and evaluating the impact of vector control intervention campaigns on these diseases.

## Supporting information

S1 Fig Location of the six districts participating in the bed net distribution campaign, with the two districts chosen for the household survey highlighted in blue. (PDF)

S2 Fig Absolute antibody response to three *P*. *falciparum* antigens in community members sampled during household surveys in Nacala-a-Velha and Mecubúri Districts, Northern Mozambique, combining data from both surveys 2013–2014. Red line indicates cut-off used to determine seropositivity. MFI: Median Fluorescent Intensity.
(PDF)

S3 Fig Absolute antibody response to *P*. *ovale*, *P*. *malaria*, *and P*. *vivax* MSP-1_19_ antigens in community members sampled during household surveys in Nacala-a-Velha and Mecubúri Districts, Northern Mozambique, combining data from both surveys 2013–2014. Red line indicates cut-off used to determine seropositivity. MFI: Median Fluorescent Intensity.
(PDF)

S4 Fig Absolute antibody response to three lymphatic filariasis antigens and the *Strongyloides* NIE (control) antigen in community members sampled during household surveys in Nacala-a-Velha and Mecubúri Districts, Northern Mozambique, combining data from both surveys 2013–2014. Red line indicates cut-off used to determine seropositivity. MFI: Median Fluorescent Intensity.
(PDF)

S1 Table Cut-offs for antigens assayed for using IgG bead-based multiplex assay on samples collected during household surveys in Nacala-a-Velha and Mecubúri Districts, Northern Mozambique, 2013–2014. (PDF)
