# Supplementary material for: Multiplex serology for impact evaluation of bed net distribution on burden of lymphatic filariasis and four species of human malaria in northern Mozambique
Source: PLoS Negl Trop Dis. 2018 Feb 14;12(2):e0006278. doi: 10.1371/journal.pntd.0006278 (PMC5854460; doi:10.1371/journal.pntd.0006278)
Supplement: S1 Fig — (PDF) [file pntd.0006278.s001.pdf]

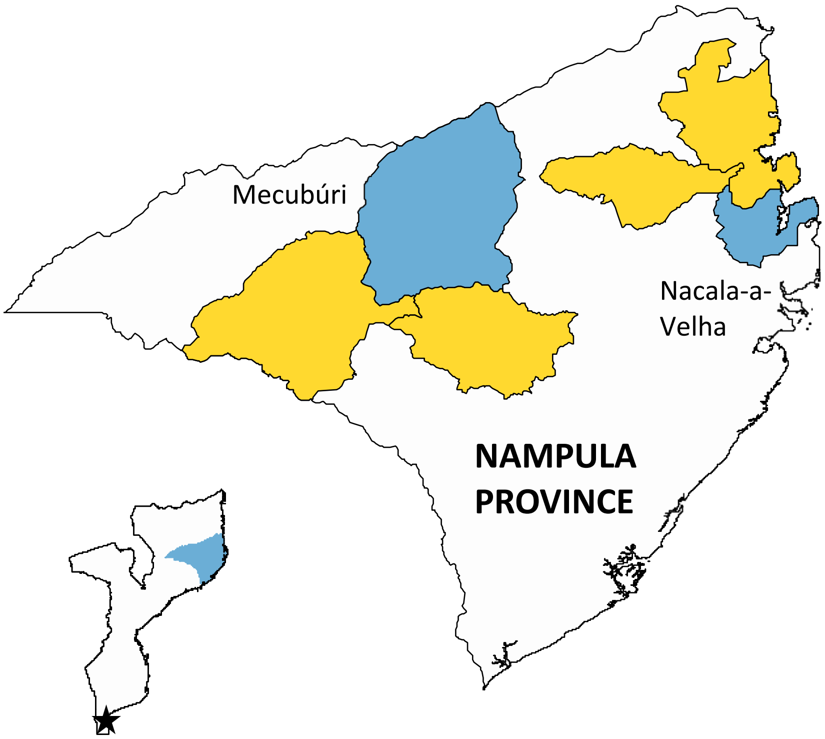

S1 Figure. Location of the six districts participating in the bed net distribution campaign, with the two districts chosen for the household survey highlighted in blue.
