# Supplementary material for: Multiplex serology for impact evaluation of bed net distribution on burden of lymphatic filariasis and four species of human malaria in northern Mozambique
Source: PLoS Negl Trop Dis. 2018 Feb 14;12(2):e0006278. doi: 10.1371/journal.pntd.0006278 (PMC5854460; doi:10.1371/journal.pntd.0006278)
Supplement: S3 Fig — Red line indicates cut-off used to determine seropositivity. MFI: Median Fluorescent Intensity. (PDF) [file pntd.0006278.s003.pdf]

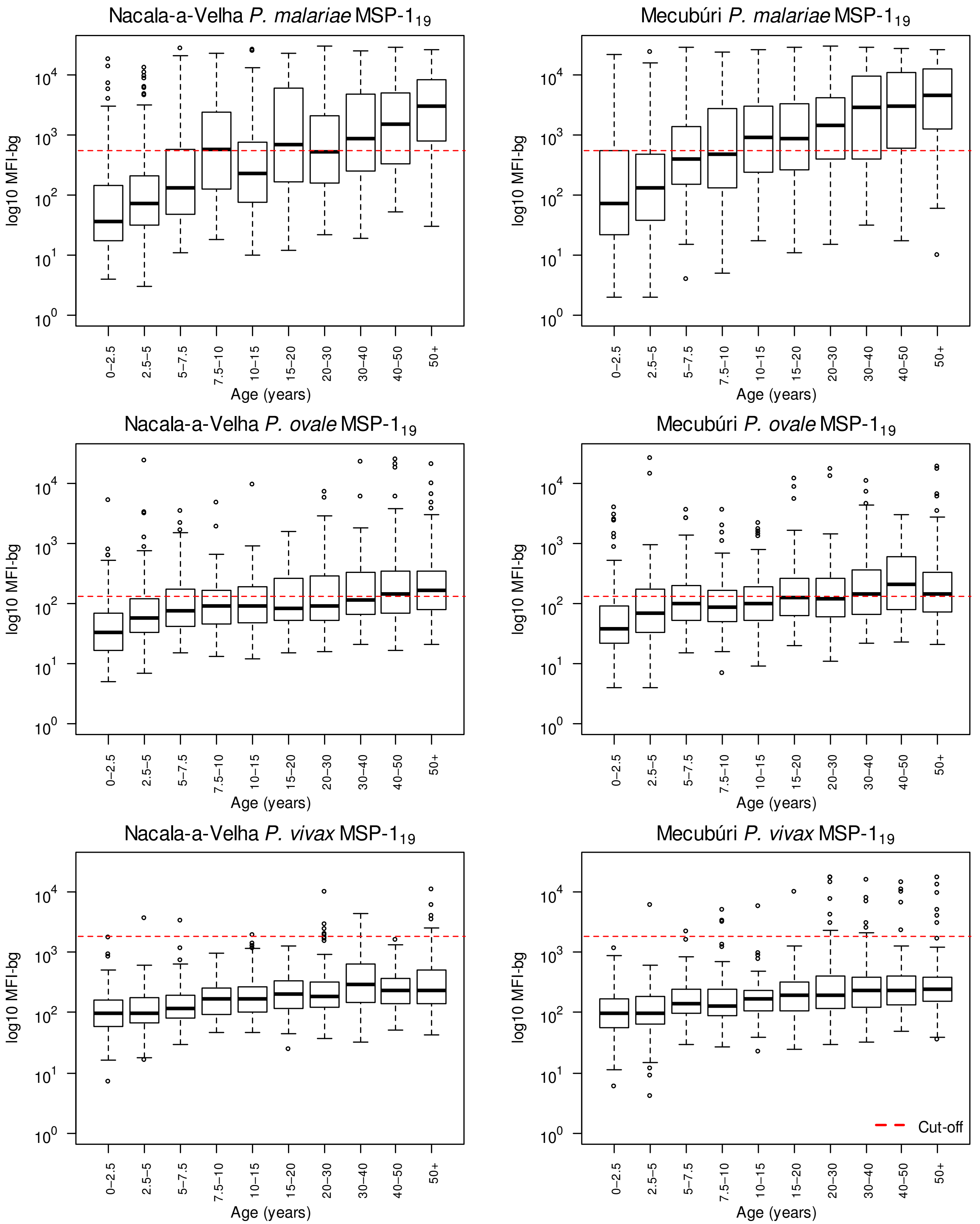

S3 Figure. Absolute antibody response to *P. ovale, P. malaria, and P. vivax* MSP-1_{19} antigens in community members sampled during household surveys in Nacala-a-Velha and Mecubúri Districts, Northern Mozambique, combining data from both surveys 2013–2014. Red line indicates cut-off used to determine seropositivity. MFI: Median Fluorescent Intensity.
