# Supplementary material for: Multiplex serology for impact evaluation of bed net distribution on burden of lymphatic filariasis and four species of human malaria in northern Mozambique
Source: PLoS Negl Trop Dis. 2018 Feb 14;12(2):e0006278. doi: 10.1371/journal.pntd.0006278 (PMC5854460; doi:10.1371/journal.pntd.0006278)
Supplement: S1 Table — (PDF) [file pntd.0006278.s005.pdf]

S1 Table. Cut-offs for antigens assayed for using IgG bead-based multiplex assay on samples collected during household surveys in Nacala-a-Velha and Mecubúri Districts, Northern Mozambique, 2013–2014

Antigen	Cut-off (MFI-bg)
P. falciparum MSP-1_{19}	615
P. falciparum CSP	1767
P. falciparum LSA-1	236
P. malariae MSP-1_{19}	538
P. ovale MSP-1_{19}	131
P. vivax MSP-1_{19}	1869
W. bancrofti Wb123	188
B. malayi Bm14	271
B. malayi Bm33	619
S. stercoralis NIE (Control)	967

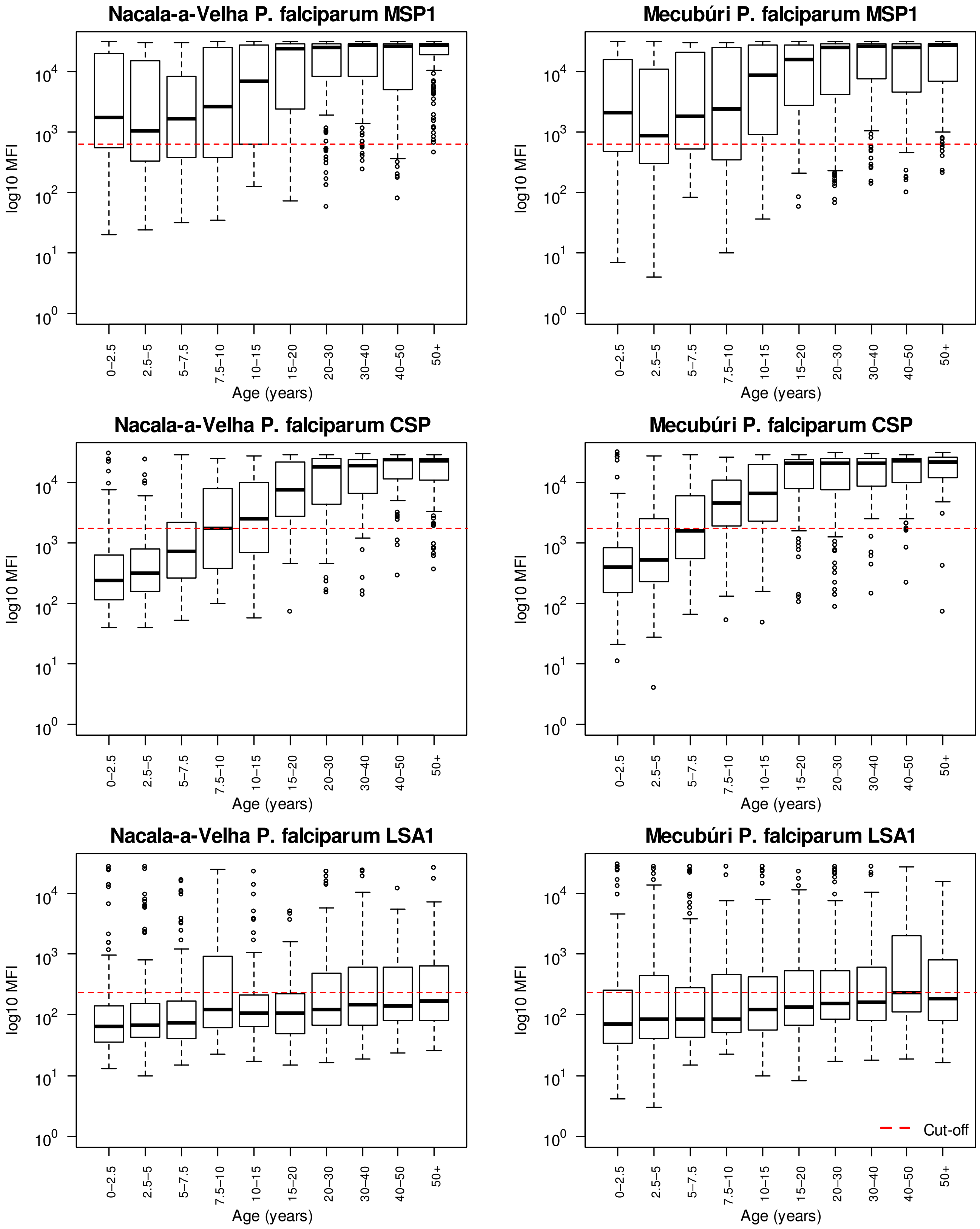

Supplemental Figure S2. Absolute antibody response to three *P. falciparum* antigens in community members sampled during household surveys in Nacala-a-Velha and Mecubúri Districts, Northern Mozambique, combining data from both surveys 2013–2014. Red line indicates cut-off used to determine seropositivity. MFI: Mean Fluorescence Intensity.

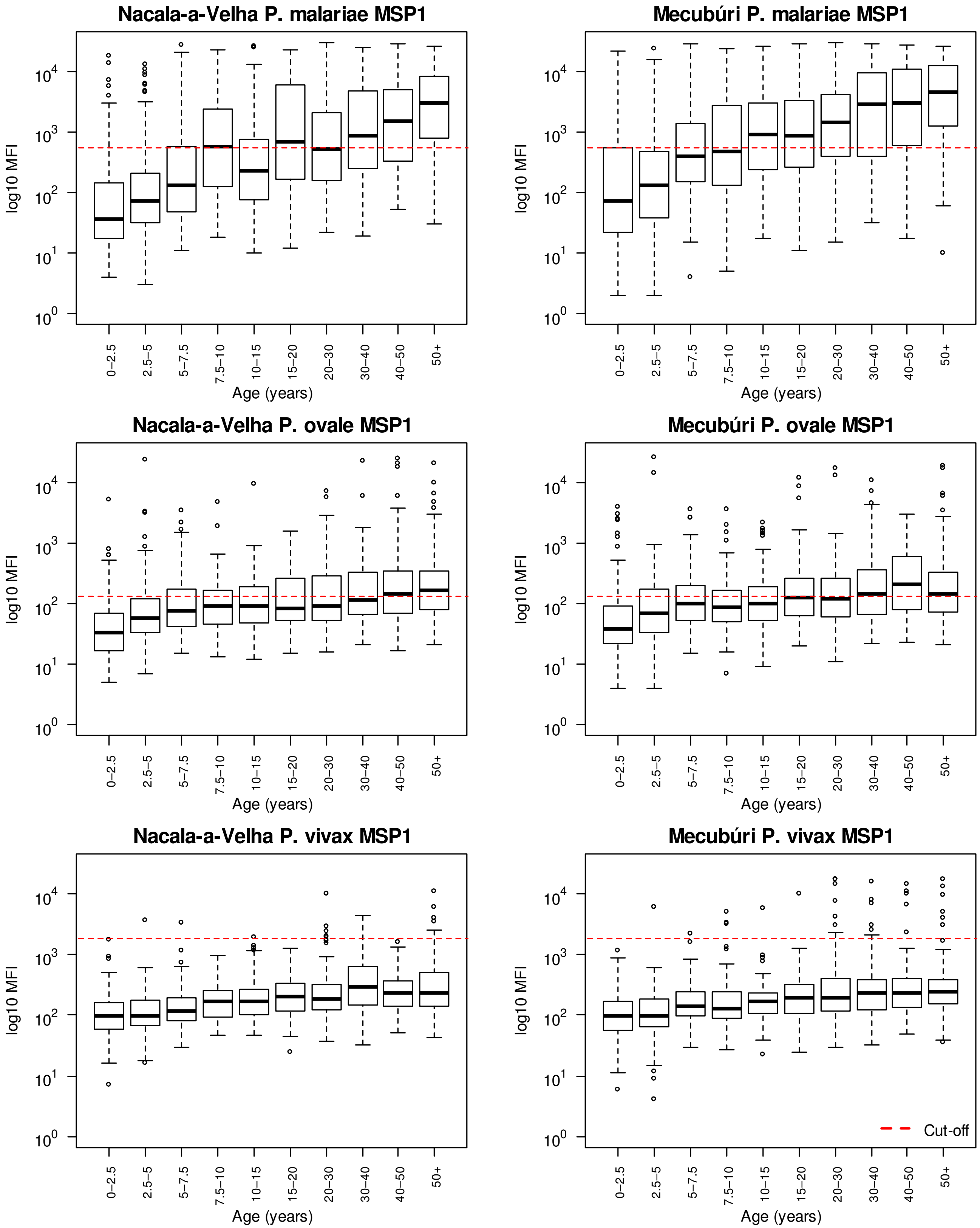

Supplemental Figure S3. Absolute antibody response to *P. ovale, P. malaria, and P. vivax* $MSP1_{19}$ antigens in community members sampled during household surveys in Nacala-a-Velha and Mecubúri Districts, Northern Mozambique, combining data from both surveys 2013–2014. Red line indicates cut-off used to determine seropositivity. MFI: Mean Fluorescence Intensity.

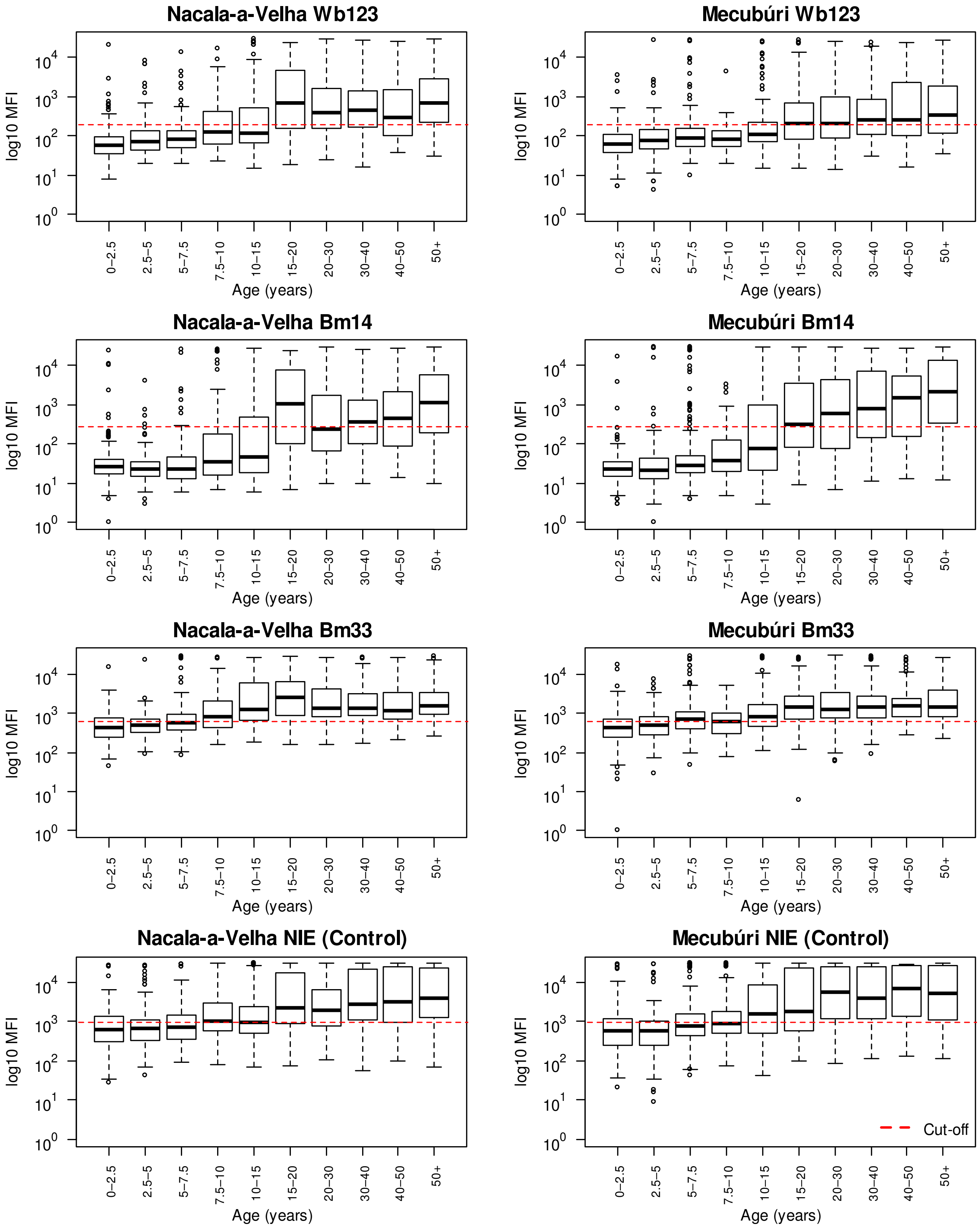

Supplemental Figure S4. Absolute antibody response to three lymphatic filariasis antigens and the strongyloides NIE (control) antigen in community members sampled during household surveys in Nacala-a-Velha and Mecubúri Districts, Northern Mozambique, combining data from both surveys 2013–2014. Red line indicates cut-off used to determine seropositivity. MFI: Mean Fluorescence Intensity.
